# Comparative study measuring optic nerve sheath diameter by transorbital ultrasound in healthy women, pregnant women and pregnant with preeclampsia / eclampsia

**DOI:** 10.1186/2197-425X-3-S1-A992

**Published:** 2015-10-01

**Authors:** J Ortega, EG Urias, C Arteaga

**Affiliations:** Instituto Mexicano del Seguro Social, Critical Care, Culiacan, Mexico; Hospital General de Culiacan, Telerobotica, Culiacan, Mexico; Centro de Investigación y Docencia en Ciencias de la Salud, Anesthesiology, Culiacan, Mexico; Centro de Investigación y Docencia en Ciencias de la Salud, Culiacan, Mexico

## Introduction

Preeclampsia / eclampsia is a potentially serious disease associated with maternal complications, including neurological. In patients with increased intracranial pressure, the diameter of the optic nerve sheath increases because of its close association with the flow of cerebrospinal fluid. Her measurements using ultrasound transorbital have shown correlation with increased intracranial pressure. 20% of patients with preeclampsia, the diameter of the optic nerve sheath consistent reaches intracranial pressure values above 20 mmHg.To compare the diameter of the optic nerve sheath transorbital measured by ultrasound between healthy women, pregnant women and pregnant women with preeclampsia / eclampsia.

## Objectives

To compare the diameter of the optic nerve sheath transorbital measured by ultrasound between healthy women, pregnant women and pregnant women with preeclampsia / eclampsia.

## Methods

Cross-sectional, multicenter study. 3 groups were included: Group1: healthy women. Group2: women with pregnancy. Group 3: women with preeclampsia / eclampsia. We obtained urine protein, serum creatinine and platelets, blood pressure, related symptoms. Diameter 3 mm behind the eyeball and an axis perpendicular to the optic nerve was measured. Three measurements of each eye were made, averaging them to give a mean to minimize the variability of the measurement.

## Results

60 patients, 20 in each group. The diameter of the optic nerve sheath was higher with statistical significance (p < 0.05) for both eyes in patients with preeclampsia / eclampsia. In group 3, 20% in the right eye and 25% in the left eye had a diameter of optic nerve sheath above 5.0 mm

## Conclusions

Pregnant patients with the diagnosis of preeclampsia / eclampsia had diameters larger than the optic nerve sheath compared with women with normoevolutivos pregnancies and healthy women. In this sense, measurement transorbital DVNO by ultrasound appears as a new promissory tool, affordable, accessible and non-invasive evaluation and timely care of patients with preeclampsia / eclampsia to rule elevated intracranial pressure.

**Table 1:** Table 1

		Right eye	Left eye
Sistolic arterial tensión	Pearson correlation Sig. Bilateral	0.455(**) 0.000	0.512(**) 0.000
Diastolic arterial tensión	Pearson correlation Sig. Bilateral	0.383(**) 0.003	0.477(**) 0.000

*[Results]*
